# A Sniff Away From Death: A Rare Case of Cocaine-Induced Triple Vessel Coronary Artery Disease in a 41-Year-Old Male Patient

**DOI:** 10.7759/cureus.40707

**Published:** 2023-06-20

**Authors:** Neel N Patel, Karan B Bhanushali, Heena K Asnani

**Affiliations:** 1 Internal Medicine, New York Medical College/Landmark Medical Center, Woonsocket, USA; 2 Medicine, Byramjee Jeejeebhoy (BJ) Medical College, Ahmedabad, IND; 3 Internal Medicine, Roger Williams Medical Center, Providence, USA; 4 Medicine, Rural Medical College, Pravara Institute of Medical Sciences, Loni, IND; 5 Internal Medicine, Rural Medical College, Pravara Institute of Medical Sciences, Loni, IND

**Keywords:** st-elevation mi, multivessel disease, cardiogenic shock, acute coronary syndrome, cocaine-induced myocardial infarction, cocaine-induced coronary disease, multivessel cad, cocaine

## Abstract

Cocaine-associated coronary artery disease and ST-segment elevation myocardial infarction (STEMI) have been well described in the literature. However, very few cases of cocaine-induced multivessel coronary artery disease have been reported. We report a very rare case of cocaine-associated triple vessel coronary artery disease in a 41-year-old male patient. The patient underwent urgent catheterization that revealed occlusion of his proximal left anterior descending artery (LAD), mid-circumflex artery, and right coronary artery with angioplasty and stent placement. His hospitalization course was complicated by cardiogenic shock, shock liver, acute renal failure, and sepsis.

## Introduction

Cocaine is the most commonly used central nervous system (CNS) stimulant. Reportedly, 11.4% of young adults have used cocaine at least once [1]. Cocaine is known to cause arrhythmias (through Na and K channel blockade), acute hypertension and coronary artery spasm (by norepinephrine reuptake inhibition, increased endothelin-1, and nitric oxide synthase inhibition), platelet aggregation, coronary artery disease, acute myocardial infarction, and cardiomyopathy (through nonischemic myocardial depression, catecholamine toxicity, and left ventricular hypertrophy) [2].

## Case presentation

**Figure 1:**
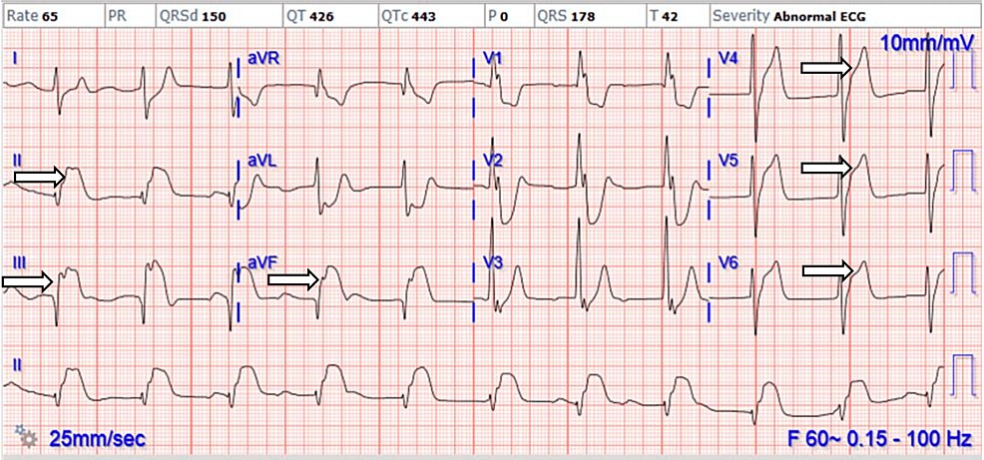
Initial EKG on admission $\Rightarrow$Acute inferolateral myocardial infarction Complete AV block with wide QRS complexes, right bundle branch block, and left posterior fascicular block EKG: electrocardiogram, AV: atrioventricular

A 41-year-old male was brought to the emergency department by emergency medical services (EMS) for a suspected drug overdose after being found unresponsive. He was given 2 mg of naloxone by the EMS. He was intermittently yelling and thrashing and then spontaneously falling asleep and was unable to provide reliable history. He was placed in restraints and medicated with diphenhydramine, haloperidol, and lorazepam. An initial evaluation in the emergency department (Figure 1) revealed bradycardia with a heart rate of 60 beats/minute and electrocardiogram (EKG) suggestive of right bundle branch block (RBBB), left posterior fascicular block (LPFB), and acute inferolateral infarct. The urine drug screen was positive for cocaine and fentanyl. High-sensitivity (HS) troponin I level was 5,917 (reference range: <76 ng/L). Code ST-segment elevation myocardial infarction (STEMI) was activated for the acute inferolateral STEMI.

**Figure 2:**
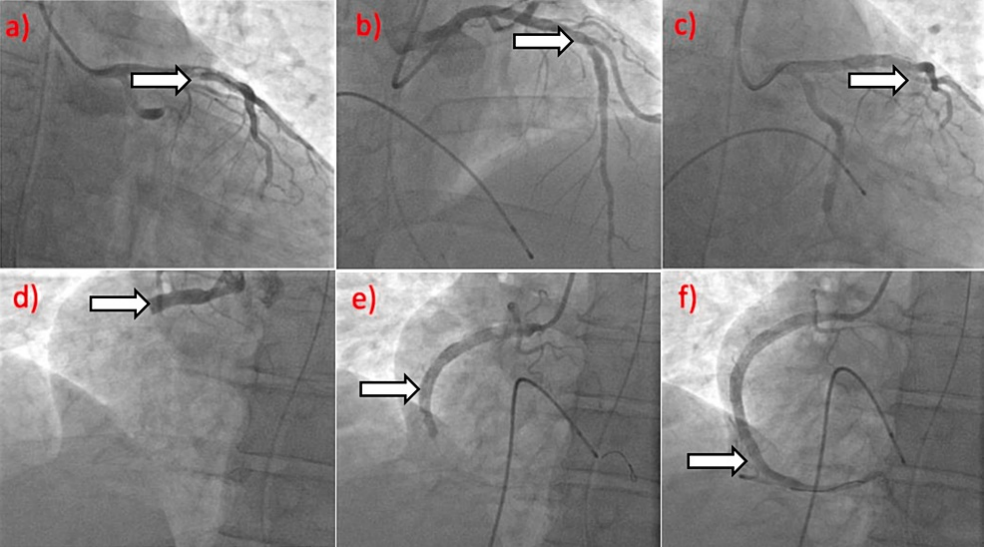
Results from the cardiac catheterization (a) $\Rightarrow$Ruptured plaque and thrombus in the proximal LAD with 90% stenosis, vasospasm, and diffuse disease in the distal LAD with 80% stenosis, and mid-vessel occlusion in the circumflex artery (b) $\Rightarrow$Successful balloon angioplasty and Onyx drug-eluting stent placement to the proximal LAD with TIMI 3 flow (c) $\Rightarrow$Successful balloon angioplasty and Onyx drug-eluting stent placement to the mid-circumflex artery with TIMI 2 flow (d) $\Rightarrow$The right coronary artery was an extensively large caliber dominant vessel with complete proximal, mid, and distal thrombotic occlusion (e) $\Rightarrow$Successful penumbra thrombectomy of the right coronary artery and intracoronary TPA infusion followed by balloon angioplasty and drug-eluting stent to the proximal right coronary artery (f) $\Rightarrow$Drug-eluting stent placement to the mid and distal right coronary artery using two overlapping stents; drug-eluting stent to the distal right coronary artery with TIMI 2 flow LAD: left anterior descending artery, TPA: tissue plasminogen activator

The patient was intubated for airway protection and underwent urgent cardiac catheterization. During the cardiac catheterization (Figure 2), left ventricular end-diastolic pressure (LVEDP) was 20 mmHg. The left main coronary artery was a large vessel without disease, and the left anterior descending artery (LAD) was a large, transapical vessel with ruptured plaque and thrombus in the proximal LAD with 90% stenosis. There was vasospasm and diffuse disease in the distal LAD of 80% severity. Successful balloon angioplasty and stent placement were performed to the proximal LAD utilizing a 4 × 22 mm Onyx drug-eluting stent (DES) deployed at a high atmosphere with 0% residual stenosis and thrombolysis in myocardial infarction (TIMI) 3 flow. Successful balloon angioplasty with drug-eluting stent (DES) placement to the mid-circumflex artery using 3.5 × 28 mm Onyx DES deployed at a high atmosphere with 0% residual stenosis and TIMI 2 flow. The right coronary artery was an excessively large caliber vessel with proximal and mid-distal thrombotic occlusion. Successful penumbra thrombectomy of the right coronary artery as well as 10 mg of intracoronary tissue plasminogen activator (TPA) infusion was done followed by balloon angioplasty and DES to the proximal right coronary artery with 5 × 30 mm Onyx DES, which dilated to 5.5 mm. A drug-eluting stent was placed in the mid and distal right coronary artery with a 2.75 × 34 mm Onyx drug-eluting stent. At the completion of the procedure, there was persistent ST-elevation with TIMI 2 flow.

He was given 3 L of IV normal saline during the procedure, maintained at normal mean arterial pressure (MAP) using norepinephrine and dopamine infusions, and sedated using 8 mg of midazolam and 100 mcg of fentanyl together with propofol after receiving paralytic for intubation.

He was transferred to the ICU maintaining a mean arterial pressure (MAP) of 60 mmHg on 10 mg of norepinephrine and 10 mcg of dopamine. He was sedated using propofol 5 mcg/kg/minute. In the ICU, he was found to have shock liver secondary to ischemia with aspartate aminotransferase (AST) and alanine transaminase (ALT) elevated at >2,000 IU/L. He also had acute tubular necrosis (ATN) secondary to ischemia with creatinine (Cr) of 5.5 mg/dL and blood urea nitrogen (BUN) of 71 mg/dL. He also had rhabdomyolysis with creatine phosphokinase (CPK) of >20,000 mcg/L and cardiogenic and septic shock with a WBC of 32.3 requiring pressors to maintain MAP. His IV fluids were continued to maintain perfusion. Piperacillin/tazobactam 2.25 g was continued. The pacemaker was adjusted at a sensitivity of 0.8 and demand mode at a rate of 50. He was considered for intermittent hemodialysis (IHD) versus continuous venovenous hemodiafiltration (CVVHDF) by the nephrology team for ATN and uptrending Cr. He was found to have anoxic brain injury as per neurology evaluation. He was also started on levetiracetam daily for seizure-like activity. On day 4 of admission, he remained on ventilation, with no neurological recovery. The temporary pacemaker was discontinued given no requirement in the last two days. On day 5, his creatinine had uptrended to 12.5 mg/dL, and the patient was started on hemodialysis. He had shown minimal signs of neurological recovery. From a cardiac standpoint, he was maintaining his pressure well, and he was weaned off of norepinephrine. On day 6 of admission, the patient was made “comfort measures only” as per family wishes. The patient was started on comfort care, and he expired on day 12 of the hospital stay.

## Discussion

Several pathophysiological mechanisms have been described for cocaine-induced myocardial infarction. Cocaine is found to promote endothelial dysfunction, which in turn can increase vascular permeability, platelet aggregation, and thrombosis. Cocaine is also reported to accelerate atherosclerosis in partially calcified unstable plaques and causes an increase in endothelial shear stress, which may lead to the rupture of the unstable plaque [3]. However, the most accepted mechanism for the development of cocaine-associated heart injury is coronary artery vasospasm in a state of high oxygen demand due to tachycardia and elevated blood pressure. These are accompanied by the activation of platelets and may lead to complete coronary artery occlusion [4]. Núñez et al. [5] demonstrated in their study that cumulative doses of cocaine caused significant coronary hypoperfusion in animal models. These observations cannot be attributed solely due to epicardial vasoconstriction. Cocaine-induced coronary ischemia may additionally result from microvascular constriction. Cocaine also causes direct myocardial injury, which may result in cardiogenic shock.

Establishing the incidence of myocardial infarction associated with cocaine use is a challenging task. One study reports the incidence of myocardial infarction (MI) in cocaine users presenting with chest pain to be 0.7%-6% [6]. Another multicenter trial presented similar results [7]. Mittleman et al. [8] in their retrospective study reported the rate of cocaine use to be 1% of 3,946 patients presenting with acute MI. They report that the incidence was highest during the first hour after administration and fell subsequently.

In patients with MI due to cocaine use, percutaneous coronary intervention (PCI) is recommended over fibrinolysis [9]. As per the American College of Cardiology/American Heart Association (ACC/AHA) 2012 guidelines on cocaine-associated MI, immediate coronary angiography should be performed in patients with angina and persistent ST-segment elevation after receiving nitrates and calcium channel blockers. They recommend PCI in patients with occlusive thrombus [10]. Both the 2008 and 2012 ACC/AHA guidelines recommend using bare metal stents in these patients [9,10]. It is important to note that in this cohort of patients, the risk of stent thrombosis is high [11]. This phenomenon is multifactorial and could be either from the prothrombotic effect due to continued cocaine use or failure to adhere to antiplatelets. The decision of the type of stent selection thus remains a complex medical decision. The use of fibrinolytic therapy (intracoronary and peripheral intravenous) should be limited to patients with STEMI who are unable to receive PCI [9,12]. It has been reported that the rate of complications including intracranial hemorrhage is higher in these patients [13-15].

## Conclusions

The cardiovascular morbidity and mortality related to cocaine use continue to remain a serious problem. PCI is recommended over fibrinolysis in patients presenting with MI. The clinical decision regarding the type of stent, balloon angioplasty, or thrombolysis needs to be made on a clinical judgment and case-by-case basis. Complete cessation of cocaine use remains key for secondary prevention.
